# Berlin Registry of Neuroimmunological entities (BERLimmun): protocol of a prospective observational study

**DOI:** 10.1186/s12883-022-02986-7

**Published:** 2022-12-14

**Authors:** Pia S. Sperber, Alexander U. Brandt, Hanna G. Zimmermann, Lina S. Bahr, Claudia Chien, Sophia Rekers, Anja Mähler, Chotima Böttcher, Susanna Asseyer, Ankelien Solveig Duchow, Judith Bellmann-Strobl, Klemens Ruprecht, Friedemann Paul, Tanja Schmitz-Hübsch

**Affiliations:** 1 Experimental and Clinical Research Center, Max Delbrück Center for Molecular Medicine in the Helmholtz Association and Charité Universitätsmedizin Berlin, Berlin, Germany; 2 Charité – Universitätsmedizin Berlin, Freie Universität Berlin and Humboldt-Universität zu Berlin, Experimental and Clinical Research Center, Lindenberger Weg 80, 13125 Berlin, Germany; 3 Max Delbrück Center for Molecular Medicine in the Helmholtz Association (MDC), Berlin, Germany; 4 NeuroCure Clinical Research Center, Charité – Universitätsmedizin Berlin, Freie Universität Berlin and Humboldt-Universität zu Berlin, Berlin, Germany; 5 German Center for Cardiovascular Disease (DZHK), Berlin, Germany; 6 Department of Neurology, University of California, CA Irvine, USA; 7 Department of Psychiatry and Neurosciences, Charité – Universitätsmedizin Berlin, Freie Universität Berlin and Humboldt-Universität zu Berlin, Berlin, Germany; 8 Berlin School of Mind and Brain, Humboldt Universität Berlin, Berlin, Germany; 9 Department of Neurology with Experimental Neurology, Charité – Universitätsmedizin Berlin, corporate member of Freie Universität Berlin and Humboldt-Universität zu Berlin, Berlin, Germany; 10 Department of Neuropsychiatry and Laboratory of Molecular Psychiatry, Charité – Universitätsmedizin Berlin, Freie Universität Berlin and Humboldt-Universität zu Berlin, Berlin, Germany; 11 Experimental and Clinical Research Center, Clinical Neuroimmunology Group, Lindenberger Weg 80, 13125 Berlin, Germany

**Keywords:** Multiple sclerosis (MS), Clinically isolated syndrome (CIS), Neuromyelitis optica spectrum disorder, Myelin-oligodendrocytic-glycoprotein – associated disease (MOGAD), Optic neuritis, Prospective observational study

## Abstract

**Background:**

Large-scale disease overarching longitudinal data are rare in the field of neuroimmunology. However, such data could aid early disease stratification, understanding disease etiology and ultimately improve treatment decisions. The Berlin Registry of Neuroimmunological Entities (BERLimmun) is a longitudinal prospective observational study, which aims to identify diagnostic, disease activity and prognostic markers and to elucidate the underlying pathobiology of neuroimmunological diseases.

**Methods:**

BERLimmun is a single-center prospective observational study of planned 650 patients with neuroimmunological disease entity (e.g. but not confined to: multiple sclerosis, isolated syndromes, neuromyelitis optica spectrum disorders) and 85 healthy participants with 15 years of follow-up. The protocol comprises annual in-person visits with multimodal standardized assessments of medical history, rater-based disability staging, patient-report of lifestyle, diet, general health and disease specific symptoms, tests of motor, cognitive and visual functions, structural imaging of the neuroaxis and retina and extensive sampling of biological specimen.

**Discussion:**

The BERLimmun database allows to investigate multiple key aspects of neuroimmunological diseases, such as immunological differences between diagnoses or compared to healthy participants, interrelations between findings of functional impairment and structural change, trajectories of change for different biomarkers over time and, importantly, to study determinants of the long-term disease course. BERLimmun opens an opportunity to a better understanding and distinction of neuroimmunological diseases.

**Supplementary Information:**

The online version contains supplementary material available at 10.1186/s12883-022-02986-7.

## Background and objective

The spectrum of neuroimmunological disorders is still evolving and broadening. Multiple sclerosis (MS), clinically isolated syndrome (CIS) and their less common differential diagnoses neuromyelitis optica spectrum disorders (NMOSD) are central in the field of neuroimmunology [1]. However, other clinical syndromes, such as myelin oligodendrocytic glycoprotein antibody associated disease (MOGAD) and autoimmune encephalitis gained more and more attention in recent years [2, 3]. The determinants for disease outbreak as well as variability in disease course, however, remain largely unknown. A commonality between this wide range of disorders is that the homeostasis of the immune system appears to be dysregulated, affecting structures of the central nervous system (CNS) and leading to neuroinflammation.

The majority of clinical observational studies follow patients from one isolated disease entity, and participants are included based on stringent diagnostic criteria. However, this restrictive disease specific inclusion approach may not be straightforward: firstly, because there is constant evolvement of diagnostic criteria over time and secondly, because of the inherent diagnostic uncertainty early after manifestation, such that diagnosis may change over time. Ultimately, some patients may be excluded from one study, due to violation of current diagnostic criteria, when it later becomes clear that they would have well suited into this disease population. The flipside of the coin is when patients present with similar symptoms and are subsumed among disease populations, which may in fact suffer from a quite different underlying pathology. Particularly rare disorders such as NMOSD are not well represented in clinical observational studies and we are therefore lacking comparable data.

A study from 2016 indicated that medical error and misdiagnosis ranges under the top causes for death in the United States [4]. Misdiagnosis and resulting inappropriate treatment are known problems in the field of neuroimmunology, particularly for MS [5]. A canonical example is that NMOSD was considered a severe form of MS, and remains frequently misdiagnosed to this day [6, 7]. Furthermore, what is now considered MOGAD has been regarded as an antibody-negative form of NMOSD just until recently [2, 8]. Optic neuritis, as a common clinical presentation of various neuroimmunological disorders [9–13] and overlap in magnet-resonance imaging (MRI) patterns [14, 15] illustrate the complexity to distinguish between underlying diseases. Correct diagnosis is of great importance as new treatment strategies are constantly evolving with new insights into the different pathobiological backgrounds. While there is some overlap in therapeutic approaches, therapeutic concepts differ and continue to evolve differently between disease entities, with some drugs effective in one disorder having been shown detrimental in others [6, 8, 16, 17].

In recent years it has become clear that technical advances and growing data provide a way to systematically characterize and compare diseases allowing for a better pathophysiological understanding and stratification of the respective pathology [18–20]. However, disease overarching data sufficient to serve this goal remain a rarity. The prospective Berlin Registry of Neuroimmunological Entities (BERLimmun), a conjunction of a decade’s experience from cohort studies, [21–23] follows patients with autoimmune neuroinflammatory disease and healthy participants over a long-term period using a pre-specified standard assessment protocol which is applied at dedicated study visits. A multimodal diagnostic panel provides comprehensive data, beyond those assessed in clinical routine, capturing patient perspective as well as clinical, structural, functional, immunological and metabolic factors.

### Aim

The central aim of BERLimmun is to build up a comprehensive database to (i) improve differentiation between neuroimmunological disease entities, (ii) investigate interrelations between structural and functional impairment and between internal (biomarkers) and external factors (environmental, including nutritional status and life-style) and severity of disease, (iii) identify determinants of disease progression, treatment response, and quality of life in patients with neuroimmunological disease in the long-term, (iv) build a systematic and comparable dataset for the study of less common diseases such as NMOSD and MOGAD.

A secondary aim of the study is to further improve diagnostics in neuroimmunological disorders by (i) identification of conditions or features that may affect the interpretation of disease biomarkers, (ii) the integration and evaluation of novel assessment tools, e.g. quantitative markers of motor function, questionnaires and analysis pipelines for their use in different disease populations.

## Methods

### Study design

BERLimmun is a single-center prospective observational cohort study set up and conducted at a research center affiliated to Charité – Universitätsmedizin Berlin, Germany. The site receives referrals mainly from urban area of Berlin but also nation-wide referrals specifically for the rarer disease entities NMOSD or MOGAD. Healthy control individuals are included to address deterministic questions and to establish center-specific normative data (e.g. for quantitative magnetic resonance imaging [MRI] analyses, cognitive testing or quantitative markers of motor function). The study duration is determined at 15 years of follow-up with annual in-person visits for diseased participants. Healthy participants will attend study visits only one, two, four, eight and 15 years after the baseline visit. Each study visit follows a protocol including a multimodal diagnostic panel. We retain the possibility to additionally invite patients outside the prespecified visit schedule, if a patient experiences a relapse, attack or otherwise acute disease progression, referred to as unscheduled visits.

The study is registered in the German Clinical Trial Register (DRKS00026761) and the entry is being updated upon relevant protocol modifications.

### Participants

We include female and male adult individuals with at least 18 years of age with a neuroimmunological diagnosis or syndrome as outlined in Table 1, who can give written informed consent to study participation. Study inclusion criteria are not restrictive and deliberately allow for inclusion of patients at any disease stage irrespective of treatment regimen or even without a definite diagnosis, e.g. early after first manifestation with isolated or partial syndromes. More specifically, the study focusses on the diagnoses CIS, MS, NMOSD and MOGAD. In addition, we include patients with other or even unknown diagnosis but a clinical or radiographic presentation suggestive of autoimmune neuroinflammatory disease, such as radiologically isolated syndrome (RIS), GFAP-encephalitis, autoimmune encephalitis, Susac’s syndrome, and isolated or recurrent optic neuritis or transverse myelitis. We additionally include adult female and male healthy subjects based on self-reported health status. Exclusion criteria as listed in Table 1 are applied at the time of screening while throughout the study duration we apply drop out criteria (see Table 1) guiding to evaluate whether a participant should not be followed further on, resulting in premature end-of-study. Criteria are examined and applied by study physicians and study board.

**Table 1:** Inclusion and exclusion criteria of BERLimmun (Exclusion criteria are only considered at baseline while drop-out criteria are checked at each follow-up visit)

**Inclusion criteria** - MS according to McDonald 2017 criteria [24], NMOSD according to IPND NMO Diagnostic 2015 criteria [25] OR other diagnosis of autoimmune disease with CNS involvement, including clinically or radiologically isolated syndromes, CRION or MOGAD OR self-declared healthy control participant - ≥ 18 years of age - active health insurance - competent to give written informed consent - signed consent
**Exclusion criteria** - contraindication to MRI investigation - pregnancy - relevant other disease that conflicts with study accomplishment according to investigator - inability to cooperate
**Drop-out criteria** - withdrawal of consent - non-compliance with protocol (decision by study board) - condition hindering study continuation (decision by study board)

### Assessments and outcome parameters

For an overview of all assessments please see Table 2.

#### Medical history and examinations

A medical history is taken at each visit, assessing demographics (age and sex), ethnicity, height and weight of the participant, life-style factors such as smoking, alcohol consumption, drug abuse, current symptoms and complaints including walking abilities, and the number of falls within the past 12 months. A trained physician confirms diagnosis by interview and previous records, obtains information regarding the onset of disease, history of attacks or relapses, comorbidities, full treatment information (drugs and supportive therapies), and performs a neurological examination including the Expanded Disability Status Scale (EDSS) using the Neurostatus version [26, 27]. As a modification, to rate the visual system score visual acuity is assessed by standard high contrast visual acuity testing (see Vision and Visual system for more detail). For a more specific clinical rating of gait and balance functions we additionally apply the scale for the assessment and rating of ataxia (SARA) [28, 29]. These components of the study visit covers at maximum 60 min at baseline and 50 min at follow-up visits.

#### Biospecimens

Venous blood drawings are conducted at the beginning of each visit in a fasting state and comprise sampling of EDTA-blood, serum, plasma and heparin samples, as well as PAXGene vials. Besides a clinical standard laboratory diagnostic panel (see Supplemental Material III for further details) other parts are stored for further scientific analyses: aliquoted serum and plasma samples as well as PAXGene tubes, are stored at -80 °C. Furthermore, peripheral blood mononuclear cells (PBMC) are isolated at each visit from heparinized blood samples according to standard operating procedures and stored aliquoted (5 × 10^6^ PBMC/aliquot) in liquid nitrogen. Cell-free plasma samples for the study of circulating desoxyribonuclease (DNA) are prepared from EDTA-blood tubes for cell-free isolation and stored at -80 °C. In addition, fixed whole blood samples are collected for deep immune profiling using mass cytometry (see supplementary material II for more detail). Serum samples from a subset of patients (i.e. patients with MOGAD, NMOSD and isolated syndromes) are sent in batches to a certified laboratory for measurements of aquaporin 4-(AQP4-) and myelin oligodendrocytic glycoprotein (MOG-) immunoglobuline (Ig)G, IgM and IgA for which (fixed) cell-based assays (CBA) are applied [30–32]. Some samples are additionally tested for MOG-IgG antibodies using a live CBA to increase sensitivity [33]. Other autoantibodies may be tested by commercial CBAs in specific subgroups of patients (e.g. GFAP antibodies in GFAP encephalitis patients). The total volume of blood drawing is < 100ml. Following each visit, participants collect stool samples at home using the OMNIgene® and OMNImet® GUT Kit and send them back to the study center. Gut microbiome taxonomy will be analysed by 16 S rRNA sequencing. We also plan analysis of serum and stool of relevant metabolic pathways, e.g. short chain fatty acids. Participants may opt-in for the use of further biospecimens obtained in the context of clinical routine, such as cerebrospinal fluid, biopsy or plasma obtained during plasmapheresis.

#### Nutrition and lifestyle

On each visit, participants will be asked about the adherence to specific diets and use of dietary supplements. For this, we apply a researcher-devised questionnaire from the Melbourne HOLISM cohort that is adapted to the German population [34]. Further, we implemented a questionnaire from the same research group related to type, frequency, duration and perceived effects of stress-reducing behaviors. Data on body composition is acquired by bioelectrical impedance analysis (BIA; BIACORPUS RX 4004 M, MEDICAL HealthCare GmbH) to calculate fat mass, fat-free mass, total body water, body cell mass and extracellular mass. Routine laboratory data comprise blood glucose, insulin and lipids (see Supplementary Material II). Participants’ dietary habits are collected with a web-based food frequency questionnaire (FFQ) [35]. In order to assess dietary intake on the macro- and micronutrient level in close proximity to stool sampling, we will implement a web-based 24 h recall [36, 37]. Both instruments are filled in remotely after each visit. With optional consent, a subgroup of participants will wear a sensor for continuous glucose monitoring for 14 days for in-depth evaluation of glucose variability (Freestyle Libre Pro, Abbott) [38] and undergo abdominal MRI for the quantification of visceral fat. Rationale of such investigation is recent evidence that abdominal fat is prevalent in MS and associated with inflammation [39] and that increased body fat is associated with adverse disease outcomes also in NMOSD [40].

#### Patient reported outcomes

Participant’s experience of health and disease is captured by a set of patient-reported outcome measures (PROMs). These cover disease related symptoms of relevance (fatigue, depression, pain, cognition), body function performance (spasticity, walking function, balance) and domains of general health and quality of life. Although some disease-specific instruments have only been validated in MS, they are here applied for all disease entities for comparability and in absence of more disease specific PROMs for rare syndromes.

Participants report about the impact of spasticity using spasticity numeric rating scale (NRS) 0-10 [41]. The duration of leisure-time physical activities is reported by the Godin-Shephard Leisure-Time Physical Activity Questionnaire (GSLTPAQ) which can be used to classify participants in physically active and insufficiently active [42]. We use patient determined Disease Steps (PDDS), which has been validated as a nine-step self-evaluation tool of physical disability in MS [43]. As a patient-based measure of walking abilities we use the 12-item MS walking scale (MSWS-12), [44] and use the Activities-specific Balance Confidence (ABC-) Scale to capture participant’s experience of balance problems [45]. We measure the impact of fatigue on typical daily tasks using the fatigue severity scale (FSS) [46]. Fatigue is additionally assessed with the fatigue scale for motor and cognitive functions (FSMC) [47]. Participant’s experience of cognitive functions is captured by the patient-reported outcomes measurement information system (PROMIS) cognitive abilities [48]. Next to a brief pain anamnesis we use the brief pain inventory (BPI) [49] and the painDETECT questionnaire, which has been proposed to be more sensitive to neuropathic pain [50]. We use Beck’s depression inventory version II (BDI-II), which has shown good validity to capture depression symptoms in MS [51]. We use the Hamburg Quality of Life Questionnaire in Multiple Sclerosis (HAQUAMS), as disease specific measure of quality of life in MS [52]. The study protocol also includes the PROMIS – general health to validate this 10-item global health PROM in a German MS population which facilitates comparison across diseases and populations [53]. Questionnaires are completed on site via tablet with 30 min allocated to completion.

#### Quantitative assessments of motor functions

Two stopwatch-tests of the multiple sclerosis functional composite (MSFC) are applied as simple quantitative assessment of motor functions, [54] namely the Timed 25-Foot Walk Test (T25FW) and the 9-Hole Peg Test (9HPT) [55]. Further, handgrip force is measured in kg by a hand-held dynamometer with the maximum value out of three trials for each hand used as standard parameter [56]. Based on previous pilot studies, [57–59] a short motor assessment protocol (PASS-MS, see Supplemental Material I for details) is applied as a novel quantitative marker of motor functions: participants perform a set of ten short motor tasks according to standardized operator instructions in front of a consumer camera with infrared sensing technology (RGB-depth camera Microsoft Kinect™, Microsoft, Redmond, WA, USA) using a custom-script user interface and data analysis tools (Motognosis Labs, Motognosis GmbH, Berlin, Germany). All tests are applied by trained operators who also document potential interfering factors for data interpretation along with motor recordings. A total of 30 min of the study visits are assigned to assess motor functions.

#### Cognitive assessment

The educational status and handedness of the participant is obtained by interview. Trained personnel assess the German version of the Brief Cognitive Assessment in Multiple Sclerosis (BICAMS), in accordance with test manuals. [60, 61] BICAMS is a brief clinical monitoring instrument comprising tests for cognitive domains with highest relevance for MS, namely symbol digit modalities test (SDMT) [62] assessing information processing speed, the learning trials of the California Verbal Learning Test-II (CVLT-II) [63], which is replaced in the German version by the Rey Auditory Verbal Learning Test (RAVLT) [64] (German version: Verbaler Lern- und Merkfähigkeitstest, VLMT) [65] and assesses immediate recall verbal memory and learning, and the Brief Visuospatial Memory Test Revised (BVMT-R) [66], which assesses visual immediate recall and learning. Cognitive testing takes about 15 min to conduct.

#### Vision and the visual system

Following a brief visual anamnesis, a thorough assessment of the visual system is conducted. Objective and subjective refraction is measured using a Tonoref II (Kidek, Tokyo, Japan) autorefraction device and recorded as sphere, cylinder and axis parameters. We assess the radius of the anterior coverture of the cornea with a keratometer to evaluate astigmatism. Non-contact tonometry is used to measure the intraocular pressure in mmHg to exclude eye pathologies, such as glaucoma. After refraction with best correction, 100% high contrast visual acuity measured monocularly with ETDRS charts, and low contrast visual acuity, measured monocularly and binocularly with 2,5% Sloan charts, are recorded as logMAR values and letter acuity. Visual fields are assessed including mean deviation in decibel with the Haag Streit Octopus (Haag Streit Group, Wedel, Germany). Visual evoked potentials (VEP) are examined using s RETI-port/scan 21 device (Roland Consult GmbH, Brandenburg, Germany) with gold cup electrodes placed on Oz-Fz according to the “10–20 International System”, under best-corrected vision and standardized room light and we record p100 latencies and amplitudes. We conduct two runs on each eye. Multifocal VEP and electroretinogram are optional components of the assessment. Participants receive optical coherence tomography (OCT) of the retina and optic nerve head using a Spectralis SD-OCT (Heidelberg Engineering, Heidelberg, Germany) with automatic real time (ART) function for image averaging in a normally lit room without drug-induced mydriasis. Structural damage and degenerative processes of the neuroretina are evaluated based on peripapillary ring scans (peripapillary retinal nerve fiber layer [pRNFL] thickness), macular volume scans (combined ganglion cell and inner plexiform layer [GCIPL], inner nuclear layer [INL]), [67] and optic head volume scans for optic nerve head morphometry [68]. The examination takes up to 90 min to perform.

#### Cerebrospinal magnetic-resonance imaging

At each visit an advanced cerebrospinal MRI protocol is performed consisting of cerebral 3D magnetization prepared – rapid gradient echo (MPRAGE), 3D T2 sampling perfecting with application-optimized contrasts by using flip angle evolution (SPACE), 3D fluid attenuated inversion recovery (FLAIR), 3D multi-parameter mapping (MPM), [69] 3D diffusion weighted imaging (DWI), 3D resting state functional MRI (rsfMRI) sequences. For the spinal cord, we include the following sequences: 2D sagittal short tau inversion recovery (STIR) for cervical and thoraco-lumbar levels and a 2D phase sensitive inversion recovery (PSIR) sequence for the cervical levels C2/C3 and C7/T1. Contrast enhancing agent is applied in CIS patients at baseline visits and may be applied if MRI are obtained during relapse or attack, e.g. at unscheduled visits. Standard parameters such as lesion count and lesion volume (mL) are manually defined by trained MRI-technicians. More advanced delineations such as the mean upper cervical cord area (MUCCA), [18, 70] or brain volume extraction will also be analysed. MRIs are recorded at the Berlin Center for Advanced Neuroimaging (BCAN) using two 3 Tesla Siemens Prisma scanners and 64-channel head and spinal coils. The entire imaging protocol is 90 min in length.

**Table 2:** Overview of the assessments conducted at specified visits (optional parts are given in brackets)

assessment	detail	*baseline*	*FU* patients	*FU* HC	*UV*
**schedule**		date of inclusion	annually year 1 to 15	at year 1,2,4,8,15	(x) optional (decision by study board)
**medical history & examination**					
demographics		x	x	x	(x)
medical history	diagnosis, attack/relapse history, comorbidities, current symptoms and complaints, dietary and lifestyle factors	x	x	x	x
therapies	disease modifying therapy, relapse therapy, full drug list, supportive therapies	x	x	x	x
neurological examination	EDSS SARA	x x	x x	x	(x) (x)
**biospecimens**					
blood samples	• biobanking 3 heparin (9ml each), 3 serum (10ml each), 3 EDTA (3,6 and 9 ml each), 1 PAX (2,5ml each). • clinical routine parameters • antibody testing	x	x	x	(x)
stool samples	standard 16 S rRNA sequencing, viability of bacteria, analysis of microbial metabolites	x	x	x	(x)
optional consent	use of biospecimens from clinical routine (cerebrospinal fluid, biopsy, plasma from plasmaphereses)				(x)
**nutrition and lifestyle**					
body height and weight	BMI	x	x	x	(x)
body composition (BIA)	body fat mass, fat-free mass, total body water, body cell mass, extracellular mass	x	x	x	(x)
vital signs	blood pressure, heart rate	x	x	x	(x)
dietary habits and practice of stress-reducing behavior	FFQ and HOLISM life-style assessment	x	x	x	(x)
optional consent	continuous monitoring of interstitial glucose concentrations over 14 days	(x)	(x)	(x)	(x)
**patient reported outcomes**					
questionnaires completed on site	NAS, GSLTPAQ, PDDS, MSWS-12, ABC-Scale, FSS, FSMC, PROMIS cognitive abilities, BPI, PainDETECT, BDI-II, HAQUAMS, PROMIS general health	x	x	x	(x)
web-based questionnaires (completed from home)	dietary intake (24-h-recall), FFQ	x	x	x	(x)
**quantitative assessment of motor functions**					
hand grip force	hand-held dynamometer, 3x each side	x	x	x	(x)
timed tests	T25-FW, 9-HPT	x	x	x	(x)
visuo-perceptive motion analysis PASS-MS	short walks, static balance, stand-up-and-sit, stepping in place, finger tapping, finger-nose test	x	x	x	(x)
**cognitive assessment**					
interview	handedness, education				
Brief International Cognitive Assessment for Multiple Sclerosis (BICAMS)	SDMT, VLMT, BVMT-R	x	x	x	(x)
**vision and the visual system**					
vision and the visual system	refraction, keratometry, non-contact tonometry, high- and low-contrast visual acuity, perimetry, VEP, OCT of macula and optic nerve head	x	x	x	(x)
optional consent	multi-focal VEP, electroretinogram (ERG)	(x)	(x)	(x)	(x)
**cerebrospinal magnetic-resonance imaging**					
neuroaxis MRI	• cerebral: MPRAGE, T2-SPACE, FLAIR, MPM, DWI, rsfMRI • spinal: STIR (whole spine), PSIR (C2&C3 and C7/T1)	x	x	x	(x)

*FU* Follow-up, *UV* Unscheduled visits, *HC* Healthy control individuals, *EDSS* Expanded disability status scale, *SARA* Scale for the assessment and rating of ataxia, *BIA* Bioelectrical impedance analysis, *BICAMS* Brief International Cognitive Assessment for Multiple Sclerosis, *SDMT* Symbol Digit Modality Test, *VLMT* Verbaler Lern- und Merkfähigkeitstest, *BVMT-R* Brief Visual Memory Test – Revised, *PASS-MS* Visuo-perceptive motion analysis, *NAS* Numeric rating scale measure of spasticity, *GSLTPAQ* Godin-Shephard Leisure-Time Physical Activity Questionnaire, *PDDS* Patient Determined Disease Steps, *MSWS-12* 12-item MS walking scale, *ABC-Scale* Activities-specific Balance Confidence Scale, *FSS* Fatigue Severity Scale, *FSMC* Fatigue Scale for Motor and Cognitive Functions, *PROMIS cognitive function* Patient-Reported Outcomes Measurement Information System – cognitive functions, *BPI* Brief Pain inventory, *BDI-II* Beck Depression Inventory-II, *HAQUAMS* Hamburg Quality of Life Questionnaire in Multiple Sclerosis, *FFQ* Food Frequency Questionnaire, *VEP* Visual evoked potentials, *OCT* Optical coherence tomography, *3D MPRAGE* 3D Magnetization Prepared – RApid Gradient Echo, *3D SPACE* 3D T2 sampling perfecting with application-optimized contrasts by using flip angle evolution, *3D FLAIR* Fluid attenuated inversion recovery (FLAIR), *3D MPM* 3D multi-parameter mapping, *3D DWI* 3D diffusion weighted imaging, *3D rsfMRI* 3D resting state functional MRI, *2D STIR* Short tau inversion recovery, *2D PSIR* 2D phase sensitive inversion recovery

### Sample size and power considerations

Sample size considerations are based on the estimated heterogeneity of patients, which will include the need of building subgroups (e.g. based on disease entity or disease modifying therapy), and on the other hand on the feasibility of study management. Since this study aims to address multiple research questions within and between multiple disease populations, an exact sample size calculation is not expedient. Based on current numbers of patients followed by or referred to our site, the sample size was pragmatically set to 650 patients. Based on estimated frequencies we expect approximately 300 patients with relapsing remitting MS, 80 patients with NMOSD, 40 patients with MOGAD and 230 patients with CIS and other neuroimmunological entities, as outlined by inclusion criteria. If patients drop out of the study, more patients will be recruited according to capacities.

We will additionally recruit a healthy control group of 85 individuals. This number was calculated to determine differences to diseased groups with sufficient power, based on data from several previous comparisons with relapsing remitting MS patients or NMOSD. Composition of the healthy control group will be controlled to allow for in-block matching because several outcome parameters show an age dependency, such as e.g. linear declines of brain volume or maximum gait speed.

### Randomization

No randomization is planned for this non-interventional study.

### Treatment or Intervention

The study is observational only and includes patients under any treatment as well as untreated patients. Treatment choice is at discretion of the treating physician, however, will be recorded and monitored as study data throughout the study.

### Electronic data capture, data monitoring and data quality control

Study data are stored in a Research Electronic Data Capture (REDCap) database [71]. REDCap is a secure, web-based application which features functions of custom imports of external sources, audit trails, monitoring, custom exports and dashboards. We use World Health Organization (WHO) issued ontologies to provide global standard codes for main and secondary diagnoses using the International Statistical Classification of Diseases and Related Health Problem (ICD) system [72] and for drug intake using the Anatomical Therapeutic Chemical (ATC) classification system, [73] which are regularly updated and monitored. Missing data are structured with missing data codes, keeping informative and random missing data differential. We use REDCap build-in data quality and consistency checks, e.g. for missing data tracking, data value validations or input access limitations wherever possible. A REDCap query function is used for weekly data monitoring. Technical measurements undergo pre-processing and data quality evaluations before automated import into the REDCap database. We use Labvantage® software for the management of blood samples and Phoenix PACS® to store, manage and process imaging data from OCT and MRI.

### Current status

The first patient was included on November 8th, 2021, and a total of 76 patients and 2 healthy subjects were included by May 2022.

## Discussion

The large prospective observational longitudinal study BERLimmun builds up a comprehensive, systematic and disease overarching data-base of patients with neuroimmunological disease entities over a long-term follow-up period of 15 years. Obtained data should pave the way to a better understanding of the history and disease course of neuroimmunological entities, including therapy response, to cross-compare diagnoses and facilitate their separation, to identify risk factors for disease progression and to elucidate disease mechanisms by in-depth immunological profiling and analyses.

The outstanding aspects of BERLimmun are the broad scope and the high quality of the prospectively collected data and the disease overarching approach. Many of the assessements included in the protocol are not usually examined in clinical routine (e.g. structured assessment of pain, dietary habits and biobanking including microbiome). The protocol also includes novel technologies such as cutting-edge OCT postprocessing pipelines to investigate retinal tissue damage, the instrumental assessment of motor function by visuo-perceptive computing, and advanced MRI sequences allowing to detect clinically relevant microstructural changes of the brain tissue, delineate functional aspects of the brain, identify more subtle brain tissue pathologies and specific subvolumes such as MUCCA [57, 69, 74, 75]. The extensive consideration of patient report on different aspects of disease will help to endorse the relevance of the evolving biomarkers. Further, the first application of the validated translations of the MSWS-12 and use of PROMIS questionnaires in a large German cohort of neuroimmunological disease will contribute to establish their validity and improve the understanding of these disorders from a patient perspective.

### Strengths and limitations

The study is conducted at a specialized clinical study center and local staff is trained in the conduct of studies according to the International Conference on Harmonisation of Technical Requirements for Registration of Pharmaceuticals for Human Use (ICH) good clinical practice (GCP) standards. The center has implemented a quality management approach certified according to ISO-9001. Assessors are trained and experienced in the collection of clinical study data and a second-look validation procedure is implemented for all clinical assessments. Instruments and questionnaires are validated for their use in at least subgroups of the BERLimmun patient population. Therefore, measurement error leading to information bias is thought to be minimal. However, a potential recall bias in patient reports, particularly with regard to disease history (e.g. relapses), must be considered. Patients with severe impairment are less likely to commit to study participation and are additionally more likely to drop out during follow up. Generally, the extensive study protocol conducted at annual inhouse patient visits may be cumbersome and exhausting for handicapped participants. In this respect, a standardized shorter version of study assessments per visit may be amended to allow follow-up in such cases. Still, selective inclusion, loss-to-follow up and drop-out may introduce selection bias. A thorough record of missed visits, missed assessments and drop-outs keeps the process transparent and a recruitment during early disease stages is considered to reduce the impact of this selection bias in our analysis. Confounding is thought to be minimized by advanced biostatistical techniques, such as confounder adjustment and matching, which will be accomplished by the large population size.

## Conclusion

Observational data are capable to provide reliable insights into causal relationships and are specifically important to measure treatment related outcomes in more heterogenous populations, that are not typically included in randomized control trials [76]. BERLimmun provides the set-up to triangulate existing evidence to a better understanding of neuroimmunological disorders and their interrelations. The database provides a foundation to improve the understanding of disease predictors and, ultimately, future patient care.

Items from the World Health Organization Trial Registration Data Set (Version 1.3.1.) [77]

**Table:** 

Primary registry and trial identifying number	German Clinical Trial Register DRKS00026761
Date of registration in primary registry	2. November, 2021
Secondary identifying numbers	3000358
Source(s) of monetary or material support	Charité – Universitätsmedizin Berlin
Primary sponsor	Charité – Universitätsmedizin Berlin
Secondary sponsor(s)	na
Contact for public queries	Dr. Tanja Schmitz-Hübsch
Contact for scientific queries	Dr. Tanja Schmitz-Hübsch
Public title	Berlin Registry of Neuroimmunological Entities (BERLimmun) (“Berliner Register neuroimmunologischer Erkrankungen (BERLimmun)“)
Scientific title	Berlin Registry of Neuroimmunological Entities (BERLimmun) (“Berliner Register neuroimmunologischer Erkrankungen (BERLimmun)“)
Countries of recruitment	Germany
Health condition(s) or problem(s) studied	Determinants, clinical course and outcomes of neuroimmunological disease entities (e.g. multiple sclerosis, neuromyelitis optica spectrum disorders, myeline oligodendrocytic glycoprotein antibody associated disease, clinically isolated syndrome among others)
Intervention(s)	noninterventional
Key inclusion and exclusion criteria	Inclusion: (1) MS diagnosis according to current diagnostic criteria (McDonald 2017) or NMOSD or MOGAD diagnosis according to current diagnostic criteria (Wingerchuck 2015) or other autoimmune disease affecting the central nervous system (e.g. radiographic isolated syndrome [RIS], isolated or recurrent optic neuritis, isolated myelitis, autoimmune glial fibrillary acidic protein [GFAP] encephalitis, autoimmune encephalitis, susac-syndrome, Balo’s concentric sclerosis) or a self-reported healthy participant. (2) 18 years of age or older (3) able to give written informed consent. (4) proband has health insurance. (5) written consent was given. Exclusion: (1) contraindication for magnet resonance tomography imaging (2) self-reported pregnancy (3) relevant other disease, which hinders the conduct of the study
Study type	Prospective observational study
Date of first enrolment	8. November, 2021
Target sample size	735
Recruitment status	Recruiting
Primary outcome(s)	(i) improve differentiation between neuroimmunological disease entities, (ii) investigate interrelations between structural and functional impairment and between internal (biomarkers) and external factors (environmental, including nutritional status and life-style) and severity of disease, (iii) identify determinants of disease progression, treatment response, and quality of life in patients with neuroimmunological disease in the long-term, (iv) build a systematic and comparable dataset for the study of less common diseases such as NMOSD and MOGAD.
Key secondary outcomes	improve diagnostics in neuroimmunological disorders by (i) identification of conditions or features that may affect the interpretation of disease biomarkers, (ii) the integration and evaluation of novel assessment tools, e.g. quantitative markers of motor function, questionnaires and analysis pipelines for their use in different disease populations
Ethics review	Institutional ethics committee of Charité – Universitätsmedizin Berlin (EA1/362/20)
Completion date	not applicable
Summary results	not applicable
IPD sharing statement	Yes, publication related patient data can be made accessible in de-identified form following consultation with the institutional data protection manager

.

## Supplementary Information

**Additional file 1.**

## Data Availability

Not applicable to this manuscript. Participant consent includes the consent to share data and material in the context of scientific cooperation related to the aims of this study. It further includes consent for future publication of data in de-identified form. In this case, prior consultation with the institutional data protection officer is mandatory to ensure compatibility with applicable regulations.

## Abbreviations

9HPT: 9-Hole Peg Test
ABC: Activities-specific Balance Confidence scale
ART: Automatic real time
ATC: Anatomical Therapeutic Chemical classification system
BDI-II: Beck’s Depression Inventory version II
BERLimmun: Berlin Registry of Neuroimmunological Entities
BIA: Bioelectrical impedance analysis
BICAMS: Brief Cognitive Assessment in Multiple Sclerosis
BMI: Body mass index
BPI: Brief pain inventory
BVMT-R: Brief Visuospatial Memory Test Revised
CBA: Cell-based assays
CIS: Clinically isolated syndrome
CNS: Central nervous system
CRION: Chronic relapsing inflammatory optic neuropathy
CVLT-II: California Verbal Learning Test-II
DWI: Diffusion weighted imaging
EDSS: Expanded Disability Status Scale
ETDRS: Early Treatment Diabetic Retinopathy Study
FFQ: Food Frequency Questionnaire
FLAIR: Fluid attenuated inversion recovery
FSMC: Fatigue scale for motor and cognitive functions
FSS: Fatigue severity scale
GCP: Good clinical practice
GFAP: Glial fibrillary acidic protein
GSLTPAQ: Godin-Shephard Leisure-Time Physical Activity Questionnaire
HAQUAMS: Hamburg Quality of Life Questionnaire in Multiple Sclerosis
ICD: International Statistical Classification of Diseases and Related Health Problem System
ICH: International Conference on Harmonisation of Technical Requirements for Registration of Pharmaceuticals for Human Use
IPND: The International Panel for *NMO* Diagnosis
T25FW: Timed 25-Foot Walk Test
MOGAD: Myelin oligodendrocytic glycoprotein antibody associated disease
MPM: Multi-parameter mapping
MPRAGE: Magnetization prepared – rapid gradient echo
MRI: Magnet-resonance imaging
MS: Multiple sclerosis
MSFC: Multiple sclerosis functional composite
MSWS-12: 12-item multiple sclerosis walking scale
MUCCA: Mean upper cervical cord area
NAS: Numeric rating scale measure of spasticity
NMOSD: Neuromyelitis optica spectrum disorders
NRS: Numeric rating scale
OCT: Optical coherence tomography
PASS-MS: Motor assessment protocol
PBMC: Peripheral blood mononuclear cells
PDDS: Patient determined Disease Steps
PROMs: Patient-reported outcome measures
PROMIS: Patient-reported outcomes measurement information system
PSIR: Phase sensitive inversion recovery
RAVLT: Rey Auditory Verbal Learning Test
RIS: Radiologically isolated syndrome
rsfMRI: Resting state functional magnet resonance imaging
SARA: Scale for the assessment and rating of ataxia
SDMT: Symbol digit modalities test
SPACE: 3D T2 sampling perfecting with application-optimized contrasts by using flip angle evolution
STIR: Short tau inversion recovery
VEP: Visual evoked potentials
VLMT: Verbaler Lern- und Merkfähigkeitstest (German version of Rey Auditory Verbal Learning Test)
WHO: World Health Organization

## Declarations

JBS has received speaking honoraria and travel grants from Bayer Healthcare, sanofi-aventis/Genzyme, and Biogen, in addition received compensation for serving on a Scientific Advisory board of Roche, not related to the presented work.

HGZ received research grants from Novartis and speaking honoraria from Bayer.

KR received research support from Novartis Pharma, Merck Serono, German Ministry of Education and Research, European Union (821283-2), Stiftung Charité and Arthur Arnstein Foundation, and travel grants from Guthy Jackson Charitable Foundation.

SA received a conference grant from Celgene and honoraria for lecturing from Alexion, Bayer, and Roche.
