# Supplementary material for: Berlin Registry of Neuroimmunological entities (BERLimmun): protocol of a prospective observational study
Source: BMC Neurol. 2022 Dec 14;22:479. doi: 10.1186/s12883-022-02986-7 (PMC9749207; doi:10.1186/s12883-022-02986-7)
Supplement: Supplementary file 1 — Additional file 1. [file 12883_2022_2986_MOESM1_ESM.pdf]

SUPPLEMENTAL MATERIAL

Berlin Registry of Neuroimmunological Entities (BERLimmun): protocol of a prospective observational study

Pia S. Sperber[1-5], Alexander U. Brandt[6], Hanna G. Zimmermann[1-4], Lina S. Bahr[1-3], Claudia Chien[1-3,7], Sophia Rekers[8,9], Anja Mähler[1-3], Chotima Böttcher[1-3,10], Susanna Asseyer[1-4], Ankelien Solveig Duchow[1-3], Judith Bellmann-Strobl[1-4], Klemens Ruprecht[9], Friedemann Paul [1-4,9,7] * & Tanja Schmitz-Hübsch[1-4]*

*equal contribution

[1] Experimental and Clinical Research Center, a cooperation between the Max Delbrück Center for Molecular Medicine in the Helmholtz Association and Charité Universitätsmedizin Berlin, Germany

[2] Charité – Universitätsmedizin Berlin, corporate member of Freie Universität Berlin and Humboldt-Universität zu Berlin, Experimental and Clinical Research Center, Lindenberger Weg 80, 13125 Berlin, Germany

[3] Max Delbrück Center for Molecular Medicine in the Helmholtz Association (MDC), Berlin, Germany

[4] NeuroCure Clinical Research Center, Charité – Universitätsmedizin Berlin, corporate member of Freie Universität Berlin and Humboldt-Universität zu Berlin, Germany

[5] German Center for Cardiovascular Disease (DZHK), partner site Berlin, Germany

[6] Department of Neurology, University of California, Irvine, California, USA

[7] Department of Psychiatry and Neurosciences, Charité – Universitätsmedizin Berlin, corporate member of Freie Universität Berlin and Humboldt-Universität zu Berlin, Germany

[8] Berlin School of Mind and Brain, Humboldt Universität Berlin, Berlin, Germany

[9] Department of Neurology with Experimental Neurology, Charité – Universitätsmedizin Berlin, corporate member of Freie Universität Berlin and Humboldt-Universität zu Berlin, Germany

[10] Department of Neuropsychiatry and Laboratory of Molecular Psychiatry, Charité – Universitätsmedizin Berlin, corporate member of Freie Universität Berlin and Humboldt-Universität zu Berlin, Germany

Supplemental Material I.:

description of short motor tasks applied in quantitative motor testing

The PASS-MS protocol includes stance,[1] comfortable and maximum speed walk,[2,3] tandem walk, steeping in place, stand-up-and-sit, pronator drift test, finger nose test, finger tapping, a pointing task, line tracking task and trunk stability.

Supplemental Material II.:

list of parameters routinely obtained at each visit

sodium (mmol/l), potassium (mmol/l), calcium (mmol/l), chlorid (mmol/l), aspartate transaminase (U/l), alanine transaminase (U/l), gamma-GT (U/l), cell counts, uric acid (mg/dl), creatinin (mg/dl), total bilirubin (mg/dl), glucose (mg/dl), total cholesterol (mg/dl), high density lipoproteinn (mg/dl), low density lipoprotein (mg/dl), urea (mg/dl), CRP (mg/l), HbA1c (%), thyroid gland stimulating hormone basal (mU/l), insuline (mU/l) measurements.

Supplemental Material III.:

Standard operating procedures for the processing and storage of blood biosamples

1. Liquid biopsy (cell-free plasma)

   Materials
   - 2 ml DNase-free cryovials
   - 15 ml DNase-free tubes
   - Blood collection tubes (Vacuette® EDTA)

   Procedure
   - Centrifuge 9 ml of EDTA-blood at 2,000 x g for 7 min at room temperature (RT)
   - Transfer the plasma into a new DNase-free tube (15 ml)
   - Centrifuge at 3,000 x g for 10 min at RT
   - Aliquot the cell-free plasma into 2 ml DNase-free cryovials, and store at -80°C

2. Serum collection

   Procedure
   - Blood (Vacuette ® Z Serum Sep Clot Activator) are stored at RT in the dark for 30 minutes in an upright position
   - After coagulation, blood samples are centrifuged at 2,000 x g for 10 min at RT
   - Aliquot the yellowish supernatant (serum) into cryovials, and store at -80°C

3. Plasma collection

Procedure

- Centrifuge 6 ml of EDTA-blood at 2,000 x g for 10 min at room temperature (RT)
- Aliquot plasma samples into 2 ml cryovials, and store at -80°C

4. <u>Isolation of peripheral blood mononuclear cells (PBMCs)</u>

Materials

- Biocoll (density = 1.077 g/ml)
- PBS, without Ca^{2+}/Mg^{2+}
- Washing medium (RPMI medium containing 5% fetal calf serum (FCS) and 1% HEPES)
- Freezing medium (freshly prepared, RPMI medium containing 40% FCS, 1% HEPES and 10% DMSO (Sigma #A3972)

Procedure

- Fill two 50 ml Falcon tubes with 15 ml Biocoll each
- Mix 27 ml heparinized whole blood (Vacuette® LH Lithium Heparin Sep) with 23 ml PBS
- Carefully layer 25 ml of blood mixture onto Biocoll
- Centrifuge at 790 x g at RT for 20 min (without breaking)
- Transfer the leucocyte layer (the white layer between plasma and Biocoll) into new 50 ml Falcon tube containing 50 ml PBS
- Centrifuge at 400 x g at RT for 10 min (with breaking)
- Discard the supernatant, then resuspend the cell pellet in 10 ml washing medium.
- Count the cells.
- Centrifuge at 260 x g at RT for 10 min (with breaking)
- Resuspend the cell pellet in freezing medium at the concentration of $5x10^6$ cells/ml
- Place the aliquots in Mr. Frosty™ freezing container and store at -80°C overnight
- Transfer the aliquot to liquid nitrogen tank

5. <u>Fixed-whole blood (for mass cytometry)</u>

Materials

- Proteomic stabilizer (PROT1, Smart Tube Inc.)

Procedure

- Transfer 500 µl whole blood (EDTA) into 1.5 ml Eppi
- Add 700 µl PROT1 buffer, mix and incubate at RT for 12 min
- Immediately store at -80°C

References

1. Behrens JR, Mertens S, Krüger T, et al. Validity of visual perceptive computing for static posturography in patients with multiple sclerosis. *Mult Scler J*. 2016;22(12):1596-1606. doi:10.1177/1352458515625807.
2. Behrens J, Pfüller C, Mansow-Model S, Otte K, Paul F, Brandt AU. Using perceptive computing in multiple sclerosis - the Short Maximum Speed Walk test. *J Neuroeng Rehabil*. 2014;11(1):89. doi:10.1186/1743-0003-11-89.
3. Grobelny A, Behrens JR, Mertens S, et al. Maximum walking speed in multiple sclerosis assessed with visual perceptive computing. Sakakibara M, ed. *PLoS One*. 2017;12(12):e0189281. doi:10.1371/journal.pone.0189281.
